# Understanding the natural history of selective IgA deficiency

**DOI:** 10.1016/j.jped.2025.03.002

**Published:** 2025-04-11

**Authors:** Nayara Maria Furquim Nasser, Antonio Carlos Pastorino, Thais Costa Lima de Moura, Beni Morgenstern, Mayra de Barros Dorna, Ana Paula Beltran Moschione Castro

**Affiliations:** Faculdade de Medicina, Universidade de São Paulo (HC-FMUSP), Instituto da Criança e do Adolescente, Departamento de Pediatria, Divisão de Alergia e Imunologia, Hospital das Clínicas, São Paulo, SP, Brazil

**Keywords:** Antibody deficiency, Recurrent respiratory infections, Selective IgA deficiency, Pediatrics, Allergic manifestations

## Abstract

**Objective:**

Patients with selective IgA deficiency (SIgAD) present elevated morbidity associated with infections, allergic conditions, autoimmune disorders, and neoplasms. This study aims to characterize clinical manifestations, disease progression, and laboratory findings in a cohort of pediatric patients with SIgAD.

**Methods:**

The study included patients with confirmed SIgAD and a clinical history of at least 5 years. Data encompassed clinical manifestations of the disease, patient outcomes, and laboratory findings, including IgA, IgG, IgM, IgE levels and complete blood count.

**Results:**

A total of 51 patients (1.2:1 female-to-male ratio) were included, with a median age at diagnosis of 6 years. Infections were the most common clinical manifestations of SIgAD (98 %), with pneumonia being the most frequent (94 %), followed by sinusitis (70 %). Additionally, 47 patients (92.1 %) exhibited allergic manifestations, including rhinitis or asthma. Autoimmune conditions were identified in 10 patients, predominantly thyroiditis (60 %), while neoplasms were observed in 3 patients. The sequence of disease onset revealed a natural progression, beginning with infectious diseases, followed significantly by allergic and autoimmune conditions. Elevated immunoglobulin levels (IgM or IgG) were observed in 25 patients, with hypergammaglobulinemia significantly associated with autoimmune conditions or the presence of autoantibodies (*p* < 0.05).

**Conclusions:**

SIgAD is a clinically significant condition. Understanding its natural history deepens our knowledge of the disease and helps early detection and diagnosis of comorbidities that may arise at various stages of a patient's life. Monitoring other immunoglobulin levels may offer potential biomarkers for predicting autoimmune conditions; however, larger studies are needed to validate these biomarkers.

## Introduction

The IgA present in mucosal surfaces constitutes the first line of defense against microorganisms through mechanisms that include direct pathogen neutralization, intracellular neutralization of viruses during transepithelial transport, and immune cell activation mediated by receptors.^1^ It also reduces or prevents the penetration of various allergens into the mucosal membranes where it is found.^2^

Selective immunoglobulin A deficiency (SIgAD) is the most prevalent inborn errors of immunity (IEI).^3^ SIgAD is defined by serum IgA levels below 0.07 g/L (or 7 mg/dL), with normal serum levels of IgG and IgM, in patients over 4 years of age, after excluding other causes of hypogammaglobulinemia.^4^^,^^5^ Its incidence varies depending on the ethnicity studied, estimated at 1:600 in the general population and 1:965 in Brazil.^6^ It is possible that SIgAD is underestimated due to the lack of routine screening protocols and because most carriers are asymptomatic.^7^ Symptomatic patients represent approximately 10–35 % of all SIgAD cases.^4^^,^^8^

In symptomatic individuals, there is a predominance of infectious processes, primarily affecting the respiratory and gastrointestinal mucosa, accompanied by allergic conditions such as asthma and rhinitis, as well as autoimmune disorders and neoplasms. In pediatric populations, recurrent infections represent the primary clinical manifestation, often warranting immunological evaluation;^8^ however, a definitive diagnosis of selective IgA deficiency (SIgAD) can only be confirmed through investigations conducted after the age of four in order to exclude other potential causes such as delayed immune system maturation and increased environmental exposure, which may account for the elevated infection rates.^1^^,^^4^

Although the symptoms of SIgAD are well-documented, there are few studies exploring the natural history of the disease and the potential for progression over the years. Our goal was to describe the clinical presentations of SIgAD and its progression, along with laboratory findings, in a cohort of pediatric patients.

## Methods

This is a prospective descriptive study with information collected from medical records, conducted at a reference center specializing in patients with inborn errors of immunity. The study was approved by the Local Research Ethics Committee (approval number 5.051.369, 2021).

All patients with a confirmed diagnosis of symptomatic SIgAD, from 1996 to the present, were included. IgA deficiency was defined by the presence of serum IgA levels below 0.07 g/L or 7 mg/dL, associated with normal serum levels of IgG and IgM in patients older than 4 years of age, after other causes of hypogammaglobulinemia had been excluded. All patients were required to have at least 5 years of clinical history. Patients with other inborn errors of immunity, genetic syndromes, or secondary IgA deficiency resulting from pharmacological treatments were excluded from the study.

The data extracted from the medical records comprised epidemiological information, (including date of birth, sex, and age at onset of symptoms), infection profile (type of infection, onset, and frequency of episodes), diagnosis of allergies (compatible history of asthma, allergic rhinitis, atopic dermatitis, and food allergy), autoimmune diseases (diagnosis confirmed by a rheumatologist), and neoplasms (diagnosis confirmed by an oncologist).

Laboratory analyses were conducted using data from medical records, and all tests were performed at the reference center's laboratory. Immunoglobulin A, M, and G levels were quantified via immunoturbidimetry (mg/dL), while Immunoglobulin E levels were measured using electrochemiluminescence (IU/mL). Hematological parameters including red blood cell count, hemoglobin concentration, leukocyte count, neutrophils, and lymphocytes were assessed. Autoantibodies were evaluated, including Antinuclear Antibodies (ANA), Anti-Peroxidase Antibodies (Anti-TPO), Anti-Thyroglobulin Antibodies (Anti-Tg), Rheumatoid Factor (RF). Anti-Neutrophil Cytoplasmic Antibodies (ANCA), Anti-SSA/RO, Anti-SSB/LA, and Anti-Native DNA were evaluated if ANA was positive or if there were suspicious clinical features. Detection methods included indirect immunofluorescence for ANA and ANCA, chemiluminescent immunoassay for Anti-TPO and Anti-Tg (IU/mL), immunoturbidimetry for RF, and ELISA for Anti-SSA/RO, Anti-SSB/LA, and Anti-Native DNA.

For the description of laboratory results, including hematology and immunoglobulin quantifications throughout the follow-up period, the following methodology was used: tests performed during active infectious episodes or hospitalizations were excluded. Subsequently, only tests with an interval of 1 year between each collection were selected for analysis. The following standard was set for classifications of anemia, leukopenia, leukocytosis, neutropenia, neutrophilia, lymphocytosis, lymphopenia, increased or decreased IgG, IgM: the majority (> 50 %) of the tests analyzed were outside the normal range described in the reference values for each test according to the patient's age.

The progression of clinical manifestations was described based on the age of onset of each listed condition: infections, allergic diseases, autoimmunity, and neoplasms. The Kruskal-Wallis test, followed by the Dwass-Steel-Critchlow-Fligner post-hoc test, was employed to determine if there were significant differences in the time of onset among the various manifestations. A *p*-value of <0.05 was considered statistically significant. Additionally, a Venn diagram was used to evaluate the overlap of clinical manifestations among the patients.

The correlation between laboratory values and clinical manifestations was evaluated using the Chi-Square and Fisher's exact tests. The levels of IgG, IgM, and IgE were analyzed in relation to the presence or absence of cardinal symptoms in patients with SIgAD, including infections, allergies, autoimmune diseases, and neoplasms.

## Results

**Figure 1:**
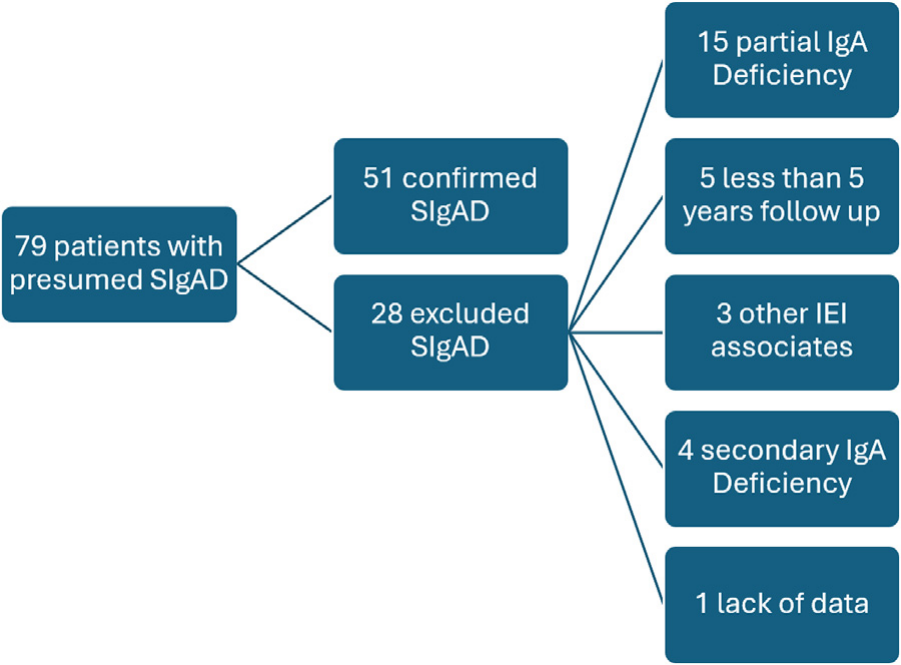
Screening design. Flowchart of patient selection.

Of the 79 patients referred to the investigation of IEI with an initial presumptive diagnosis of SIgAD, 51 met the inclusion criteria (Figure 1). The 51 patients (1.2F:1 M) were followed for an average of 10 years with a standard deviation of ± 2.8 years (ranging from 5 to 16 years). The median age at the time of SIgAD diagnosis was 6 years (ranging from 4 to 13 years), and the median age at the onset of the first symptom was 1 year. Consanguinity was reported in two families, and only one of the cases was associated with other instances of SIgAD. A family history of SIgAD was observed in eight patients.

The most frequent clinical manifestations were infectious, with a notably early onset, particularly involving respiratory infections (Table 1). More than half of the patients (66 %) experienced involvement of multiple infectious sites during the follow-up period. Respiratory allergies, especially rhinitis, were highly prevalent (97.8 %), with asthma occurring in 31 patients (67 %). An association between respiratory allergies and airway infections was observed in 45 patients (88.2 %).

**Table 1:** Clinical features of confirmed of SIgAD patients.

Clinical features				
Symptoms related to:	N ( %)	Median age at onset (years) (min–max)	Diseases	N (%)
Infection	50 (98.0)	1.0 (0.1–6)	Pneumonia	47 (94.0)
			Sinusitis	35 (75.0)
			Otitis	27 (54.0)
			Gastrointestinal	27 (54.0)
			Tonsillitis	22 (44.0)
			UTI^a^	13 (26.0)
			Impetigos	4 (8.0)
			Parotitis	2 (4.)
			Viral meningitis	2 (4.0)
			Ostomastoiditis	1 (2.0)
			Sepsis	1 (2.0)
			Osteomyelitis	1 (2.0)
			Skin abscess	1 (2.0)
Allergies	47 (92.1)	5.0 (0.1–15)	Rhinitis	46 (97.8)
			Asthma	30 (63.8)
			Atopic dermatitis	9 (19.1)
			Food allergy	3 (6.3)
Autoimmunities	10 (19.6)	8.0 (4.0–13)	Tideoiditis	6 (60.0)
			DM1^b^	1 (10.0)
			Rheumatic fever	1 (10.0)
			Celiac disease	1 (10.0)
			ITP^c^	1 (10.0)
			JIA^d^	1 (10.0)
			Vitiligo	1 (10.0)
			Grave’ disease	1 (10.0)
			SLE^e^	1 (10.0)
Neoplasms	3 (5.8)	9 (1.6–15)	Langerhans histiocytosis	2 (66.6)
			Hodgkin's lymphoma	1 (33.3)

a Urinary tract infection.

b Type 1 diabetes mellitus.

c Idiopathic thrombocytopenic purpura.

d Juvenile idiopathic arthritis.

e Systematic lupus erythematosus.

Autoimmune diseases were concomitant in two of the eight affected patients. The presence of autoantibodies without clinical manifestations was detected in 31 patients (60.7 %), beginning at 3 years of age, with a median age of antibody detection at 9 (ranging from 3 to 15 years). The most common autoantibodies were antinuclear antibodies (21 patients, 67.7 %), followed by antithyroglobulin and antithyroid peroxidase antibodies, each being detected in 12 patients (38.7 %).

**Figure 2:**
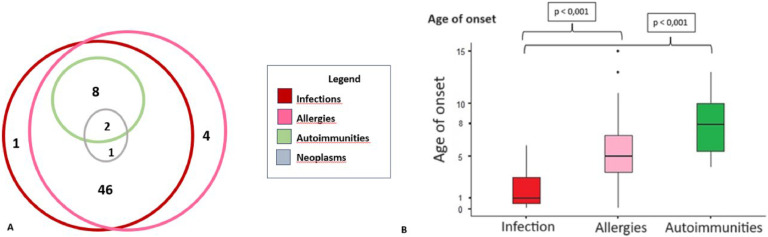
Overlapping disease and follow up. (A) Venn diagram of overlapping disease in patients with SIgAD. (B) Age-dependent disease variation in patients with IgA deficiency.

Over a period of at least five years of disease history, the ages of onset for the cardinal symptoms showed statistically significant differences, with infections being the earliest clinical manifestations of SIgAD compared to allergies and autoimmune conditions (*p* < 0.05). It was shown that the majority of patients had more than one associated clinical manifestation (Figure 2). Allergies and infections were the most frequently observed manifestations in this patient group. None of the patients presented with autoimmune diseases in isolation. Among the three patients with neoplasms, two also presented autoimmunity, infections, and allergies, while the third patient only experienced infections and allergies.

During the follow-up, changes in diagnosis were observed: nine patients progressed to partial IgA deficiency at an average age of 10.6 years (ranging from 6 to 14 years), of which four normalized IgA levels over the years, with an average age of 13 years (ranging from 9 to 15 years). Two patients progressed to Common Variable Immunodeficiency (CVID), one at 12 years of age and the other at 18 years.

Laboratory tests were collected throughout the follow-up period. On average, patients underwent 9 test collections (ranging from 3 to 15 tests per patient, with a median of 9). Regarding blood counts, four patients (7.8 %) had anemia, four (7.8 %) had mild leukopenia, (3280–4600 cells/mm^3^) and none had lymphopenia.

Considering the immunoglobulin levels (IgG, IgM), 25 patients (49 %) presented values above the 97.5th percentile, indicating hypergammaglobulinemia. Among these patients, 14 (56 %) showed elevated values on more than 70 % of their samples. IgG levels were above the age-specific reference values in 20 of the 25 patients, while IgM levels were elevated in 5 of the 25 patients.

**Figure 3:**
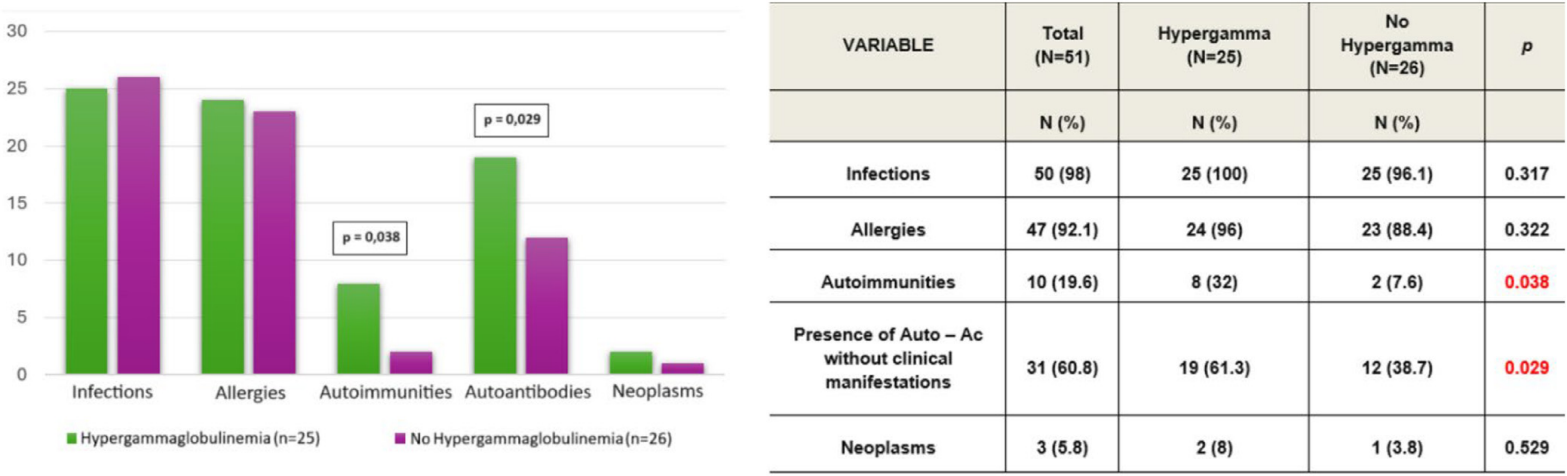
Immunoglobulins levels: correlations between hypergammaglobulinemia and clinical features in SIgAD patients (A and B).

There was a correlation between elevated levels of IgG or IgM and a higher prevalence of autoimmune diseases and autoantibodies without clinical manifestations (*p* < 0.05). However, there was no significant correlation between these elevated levels and the presence of infections or allergies (Figure 3).

On average, 4.8 (ranging from 2 to 10) IgE measurements were taken per patient during the follow-up. Of the 51 patients, 20 presented IgE levels above the age-specific reference values. However, there was no significant clinical correlation between elevated IgE levels and the presence or severity of allergies.

## Discussion

SIgAD is the most prevalent IEI, with prevalence rates among blood donors varying from 1:163 in Spain and 1:143 in the Arabian Peninsula to 1:4100 in China and 1:18,550 in Japan, suggesting a lower prevalence among Asian populations.^9^ Few studies have examined the long-term outcomes of patients with SIgAD, making our case series relevant for specialists, pediatricians, and clinicians who may encounter asymptomatic or oligosymptomatic patients with routine tests showing low IgA levels.^10^ Due to the characteristics of this cohort, the data were not compared to the normal population. However, the high number of infectious processes—more than three per patient, including pneumonia—characterizes this population as one warranting immunological investigation.

The patients included in this study showed a slight predominance of females, differing from the literature, which typically shows a male predominance, without any specific reason, probably related to the sample size.^8^ The presence of a family history of SIgAD varies across studies, and cultural aspects must be considered. In countries with a high number of consanguineous marriages, the rates of multiple cases within one family are higher than in other countries. In our study, the authors observed few consanguineous families and a low rate of family history.^11^^,^^12^

The association between IgA deficiency and infections is due to the lack of mucosal defense mechanisms, which are not fully compensated for by other immunoglobulins. IgA is the main class of immunoglobulin on mucosal surfaces and is highly effective in containing pathogens without significant tissue damage due to its protective characteristics (local non-inflammatory defense and absence of complement activation). IgA functions by blocking the adhesion and aggregation of microorganisms and may assist in the intracellular neutralization of pathogens.^13^

Upper respiratory tract infections are a common complaint in pediatrics for multiple reasons and some of these children may have SIgAD, a diagnosis that needs to be considered. In this study, infections were the most frequent manifestations, with upper respiratory tract infections being particularly prominent. In children, recurrent infections are the primary manifestations that may lead to an immunological investigation.^14^ Respiratory and gastrointestinal tract infections are the most common clinical manifestations in children with symptomatic SIgAD, occurring in 40–90 % of cases.^8^^,^^13^ Pneumonia was the most frequent complaint, and while IgA is more crucial in the upper airway, this study, conducted in a tertiary hospital, may have a bias toward more severe patients. However, the literature describes phenotypes of IgA deficiency where some patients may have more severe manifestations or may be associated with other deficiencies such as IgG2 and/or IgG3 subclasses. More severe infectious manifestations such as otomastoiditis, sepsis, and osteomyelitis were described as isolated events in this study and in literature reports.^8^^,^^11^^,^^13^^,^^14^

One limitation of this study was the use of medical records as a data source, which restricts the detail and quantification of the progression of infectious processes. Nevertheless, the scarcity of studies evaluating the natural history of SIgAD underscores the importance of these findings.

In SIgAD, although the underlying pathogenetic mechanism is not yet fully elucidated, the reduction of serum and secretory IgA can lead to colonization and penetration by pathogenic bacteria, and facilitate the passage of aeroallergens and food antigens, predisposing patients with SIgAD to a higher risk of developing allergies. The absence of IgA in mucosal surfaces may contribute to increased permeability and greater exposure to allergens, facilitating the development of allergies.^7^^,^^15^

In this study, allergic diseases significantly affected patients with SIgAD and had an early onset, around 5 years of age. The authors observed high rates of allergic rhinitis and asthma, with less pronounced rates of atopic dermatitis and food allergies, a finding consistent with previous reports.^16^^,^^17^ Allergic manifestations are possible in patients with various primary immunodeficiencies (PIDs), but a study by the World Allergy Organization (WAO) showed that SIgAD is the second most prevalent PID associated with allergies.^18^ Patients with severe asthma may be a key group for SIgAD screening, with a local study detecting a prevalence of 3.8 % among patients with severe asthma.^7^^,^^15^^,^^19^

Some studies demonstrate that SIgAD leads to the activation of compensatory mechanisms, such as the increase in secretory IgM.^20^ Another study suggests that systemic IgG and secretory IgA work together in different body compartments to limit systemic pro-inflammatory pathways. IgA mediates a homeostatic dialogue between the systemic immune compartment, although this mechanism is not yet fully understood.^21^ Our data suggests that patients with SIgAD exhibited elevated levels of IgG and IgM, along with a higher prevalence of autoimmunities and autoantibodies without clinical manifestation.

Autoimmune diseases were the third most prevalent group of conditions in our study, with significant variability in clinical presentations and with few associations between diseases. The causes of these associations were not clear, and the mechanisms may be multiple and non-exclusive. One hypothesis suggests that certain human leukocyte antigen (HLA) genes in the MHC locus on chromosome 6 may favor the development of SIgAD and autoimmune diseases, or that an unknown genetic defect could explain the association between SIgAD and autoimmunity, characterizing this phenotype of SIgAD as a monogenic disease.^22^^,^^23^

A thorough review by Odineal & Gershwin suggests that abnormalities in B cells, T cells, or cytokines in SIgAD patients may contribute to autoimmune susceptibility. The lack of IgA might contribute to a state of persistent local and systemic inflammation. Persistent inflammation leads to several negative health consequences, partly through the release of interferon IFN-γ, type I IFNs, interleukin IL-1β, IL-12, IL-17, and tumor necrosis factor-α, and may predispose immune cells to sensitize to autoantigens. Additionally, IgA interacts with cell receptors such as FcαRI to negatively regulate immune pathways, which can, in turn, propagate inflammation.^1^^,^^9^^,^^24^

Although occurring later, autoimmune diseases are notably present in patients with IgA deficiency, making them a significant manifestation to monitor and manage in the long term.^24^ A study conducted on Turkish children with SIgAD found autoimmune manifestations in 17.3 % of cases, a high percentage considering the age of this population (mean age of the study was 9.9 years ± 3.9).^24^^,^^25^ In our study, the median age of onset for autoimmune diseases was eight years, which is considered early for most autoimmune diseases diagnosed. In pediatrics, most autoimmune diseases typically occur during puberty,^26^ particularly around 16 years of age. Koskinen S, after long-term follow-up of patients with SIgAD, observed the onset of autoimmune diseases at an average age of 31 years. This finding highlights the need for long-term follow-up of patients with SIgAD to detect autoimmune manifestations and enable early diagnosis of these conditions.^1^^,^^27^

It is worth noting the high rate of autoantibodies among patients, as observed by Sarmiento and Gulez et al. In the latter study, the presence of autoantibodies was significantly higher in children with SIgAD compared to healthy control children. Although frequently, the detection of autoantibodies cannot predict whether a patient with SIgAD will develop an autoimmune disease.^28^

The follow-up of patients with SIgAD presents several challenges: they may exhibit extremely variable phenotypes, ranging from entirely asymptomatic to mild or severe infections, allergies, and autoimmune diseases.^11^ In this study, during at least five years of follow-up after the diagnosis of SIgAD, it was observed with statistical significance that the ages of onset for infections, allergies, and autoimmune diseases were variable. Yazdani et al. (2015) classified SIgAD into different phenotypes and severe and allergic ones tend to manifest in childhood, while mild infections and autoimmune phenotypes are more commonly diagnosed in midlife.^11^ Considering the progression of our patients with SIgAD, most of whom exhibited overlapping manifestations, it becomes evident that classifying them into a single phenotype is challenging. Most of our patients showed high rates of association between infections and allergies. Nearly one-fifth presented an association among all three major disease groups, revealing the complexity of SIgAD and the need for multidisciplinary and long-term follow-up.

Plebani et al. demonstrated that patients with SIgAD showed higher serum concentrations of IgG, IgM, and secretory IgM compared to controls.^29^ Secretory IgA appears to support the intestinal barrier in maintaining homeostatic levels of interaction between commensal microbes and the systemic immune system. In the absence of secretory IgA, the barrier's function is compromised, resulting in increased systemic exposure to commensal microorganisms. This can lead to aberrant humoral and systemic responses to these microbes.^30^

SIgAD in children is a relevant and common condition. Understanding the natural history of patients with SIgAD deepens the comprehension of the disease and helps early awareness and diagnosis of comorbidities that may arise at different stages of the patient's life. Regular monitoring of IgA levels is necessary, with attention to the potential for normalization; however, ongoing follow-up is crucial due to the risk of progression to other immunodeficiencies, the development of autoimmune diseases, and vigilance for neoplasms. Additionally, monitoring other immunoglobulins may aid in identifying potential biomarkers for autoimmunity, though further studies are needed to validate these biomarkers.

## Funding

This study has not received any specific grants from funding agencies in the public, commercial, or non-profit sectors. NMFN received a grant from CAPES (number 88887.806719/2023-00).

## Conflicts of interest

The authors declare no conflicts of interest.
